# Inhibition of T-Type Voltage Sensitive Calcium Channel Reduces Load-Induced OA in Mice and Suppresses the Catabolic Effect of Bone Mechanical Stress on Chondrocytes

**DOI:** 10.1371/journal.pone.0127290

**Published:** 2015-05-26

**Authors:** Padma P. Srinivasan, Ashutosh Parajuli, Christopher Price, Liyun Wang, Randall L. Duncan, Catherine B. Kirn-Safran

**Affiliations:** 1 Department of Biological Sciences, University of Delaware, Newark, Delaware, 19716, United States of America; 2 Biomedical Engineering Program, University of Delaware, Newark, Delaware, 19716, United States of America; 3 Department of Mechanical Engineering, University of Delaware, Newark, Delaware, 19716, United States of America; University of California Davis, UNITED STATES

## Abstract

Voltage-sensitive calcium channels (VSCC) regulate cellular calcium influx, one of the earliest responses to mechanical stimulation in osteoblasts. Here, we postulate that T-type VSCCs play an essential role in bone mechanical response to load and participate in events leading to the pathology of load-induced OA. Repetitive mechanical insult was used to induce OA in Ca_v_3.2 T-VSCC null and wild-type control mouse knees. Osteoblasts (MC3T3-E1) and chondrocytes were treated with a selective T-VSCC inhibitor and subjected to fluid shear stress to determine how blocking of T-VSCCs alters the expression profile of each cell type upon mechanical stimulation. Conditioned-media (CM) obtained from static and sheared MC3T3-E1 was used to assess the effect of osteoblast-derived factors on the chondrocyte phenotype. T-VSCC null knees exhibited significantly lower focal articular cartilage damage than age-matched controls. *In vitro* inhibition of T-VSCC significantly reduced the expression of both early and late mechanoresponsive genes in osteoblasts but had no effect on gene expression in chondrocytes. Furthermore, treatment of chondrocytes with CM obtained from sheared osteoblasts induced expression of markers of hypertrophy in chondrocytes and this was nearly abolished when osteoblasts were pre-treated with the T-VSCC-specific inhibitor. These results indicate that T-VSCC plays a role in signaling events associated with induction of OA and is essential to the release of osteoblast-derived factors that promote an early OA phenotype in chondrocytes. Further, these findings suggest that local inhibition of T-VSCC may serve as a therapy for blocking load-induced bone formation that results in cartilage degeneration.

## Introduction

Osteoarthritis (OA) is the most common form of arthritis and affects the whole joint, causing not only the loss of articular cartilage but also synovial inflammation, subchondral bone sclerosis and osteophyte formation. Although it is not entirely clear whether changes in subchondral bone precede cartilage changes or vice versa, it is widely accepted that subchondral bone sclerosis and remodeling are closely associated with OA development [1, 2].

Subchondral bone remodeling and its effects on the overlying cartilage may be the result of mechanotransduction, a process in which the mechanical signals encountered by the cell are converted into biochemical and cellular events [3]. Mechanical stimulation is important for skeletal integrity and it has been clearly established that routine exercise is essential to maintain normal bone mass [4]. Physical activity induces deformations of the bone matrix and shearing forces on osteoblasts and osteocytes, stimulating them to secrete various active metabolites including prostaglandins, nitric oxide, and pro-inflammatory cytokines [5, 6]. Although such metabolites are established markers of bone anabolic responses to mechanical load and are essential for stimulating bone formation, persistent secretion of soluble factors such as cytokines by subchondral osteoblasts following altered joint loading may be responsible for initiating OA-like changes in the adjacent cartilage tissue [7].

A rapid increase in intracellular calcium level is the earliest recorded biochemical response following mechanical stimulation in the osteoblasts and chondrocytes [8, 9]. Voltage-sensitive calcium channels (VSCC) have been shown to be important in altering intracellular calcium concentration in osteoblasts via calcium influx elicited by membrane depolarization [10] and are known to play an essential role in regulating intracellular processes, including cytokine signaling [11, 12]. VSCCs are multimeric protein complexes with pore forming (α1) and auxiliary (α2δ and β) subunits and are classified into high-voltage activated (long lasting or L-VSCC) and low-voltage activated (transient or T-VSCC). Based on the α1 subunit, L-VSCC is further divided into four isoforms (Ca_v_1.1, Ca_v_1.2, Ca_v_1.3 and Ca_v_1.4) and T-VSCC into three isoforms (Ca_v_3.1, Ca_v_3.2, Ca_v_3.3) [13, 14]. Interestingly, the transiently active Ca_v_3.2 T-VSCC isoform that requires weak membrane depolarization for activation is expressed by osteoblasts, osteocytes, and chondrocytes and is considered to be a major mediator of plasma membrane calcium permeability during osteogenesis [13].

Recent studies performed using a mouse model carrying a global null mutation for the *Cacna1h* gene (Ca_v_3.2 KO) display reduced bone formation, reduced subchondral bone remodeling, and diminished rate of mineral apposition following disuse-induced bone loss compared to wild type (WT) littermates [15]. Here, we postulate that the T-VSCC located in the plasma membrane of osteoblasts and/or chondrocytes mediate mechanotransduction signals and plays an essential function in the initiation of OA. To test this hypothesis, we used the Ca_v_3.2 KO mouse model carrying a null mutation for the Ca_v_3.2 α1 subunit of the T-VSCC in all tissues and induced OA using a non-invasive *in vivo* knee loading model. *In vivo* loading can induce a number of mechanical stimuli on cells present in the joint tissue including; compression, strain, hydrostatic pressure and fluid shear [16]. Here, we focused on the effects of fluid shear stress (FSS) and used an *in vitro* co-culture system to show that mechanical stimulation of osteoblasts with FSS induces the release of soluble factors that have catabolic activity on chondrocytes. To investigate downstream effects of T-VSCC activation in response to FSS in either osteoblasts or chondrocytes, we used a pharmaceutical blocker, NNC55-0396 (NNC) to selectively inhibit T-VSCC-associated calcium currents [17].

## Materials and Methods

### Ethics statement

All animal loading procedures were performed under isoflurane anesthesia as approved by the University of Delaware Institutional Animal Care and Use Committee (AUP#1170) and no adverse effect such as swelling or locomotion difficulty were observed during the entire length of the experiment. All animals were individually housed following experimental induction of osteoarthritis via *in vivo* knee loading.

### *In vivo* load induced mouse model of OA

**Fig 1:**
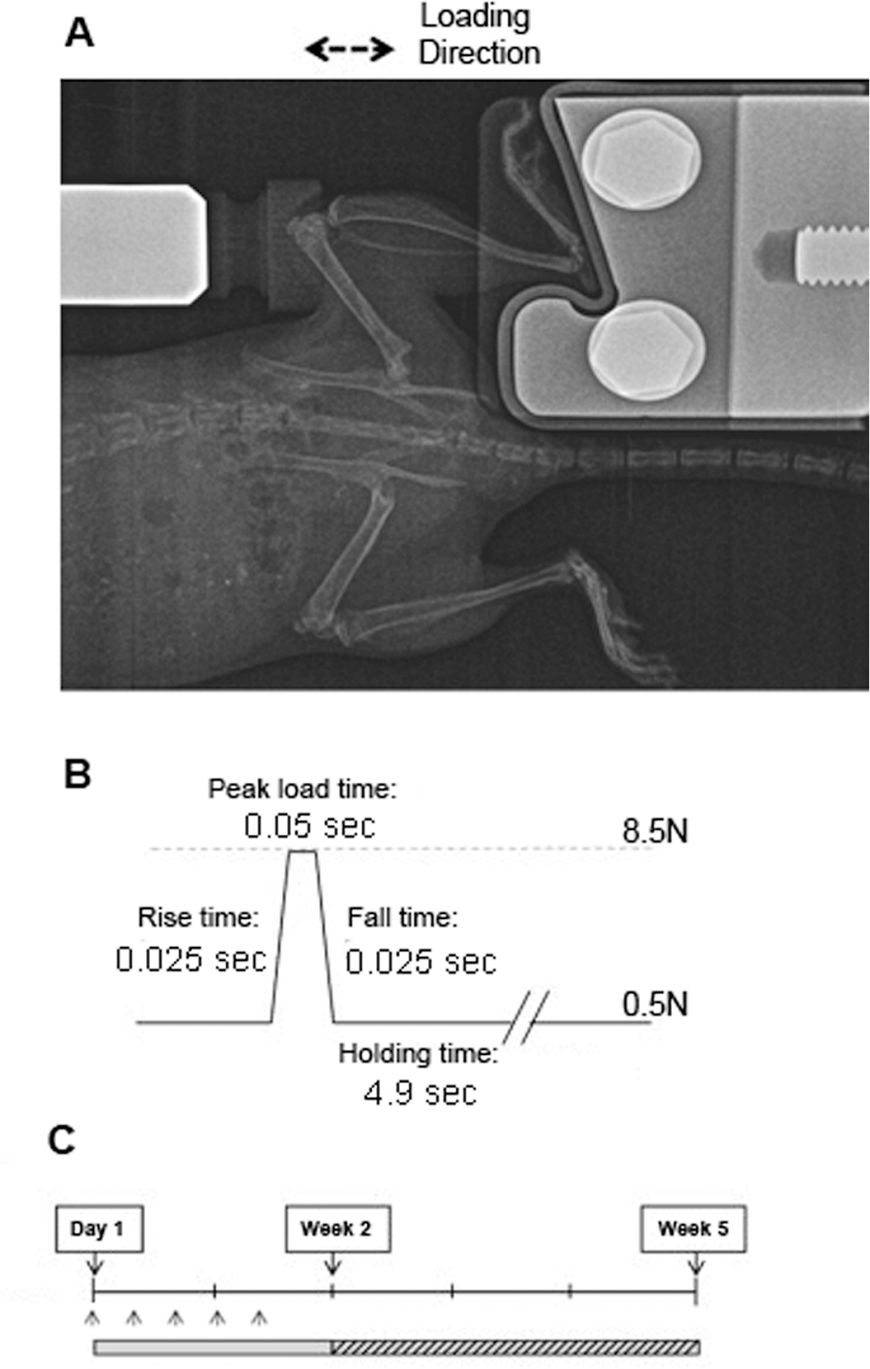
*In-vivo* loading system used to experimentally induce knee OA and compare progression of disease severity between T-VSCC and WT control knees. (A) Radiograph of mouse knee joint during loading using the Bose ElectroForce loading apparatus, (B) loading cycle waveform including a 0.025sec of rise time, 0.05sec peak load time, 0.025sec fall time and 4.9sec holding/resting time, (C) loading regimen: Five loading episodes were performed over a period of eight days followed by a 26-day period of non loading prior to sacrifice (week 5) and histological processing.

Skeletally mature 10–12 week-old male Ca_v_3.2 T-VSCC KO mouse males homozygous for a targeted null mutation in the *Cacna1h* gene and backcrossed onto the C57BL/6J inbred background (stock# 013770) were obtained from the Jackson Laboratory along with age-matched C57BL/6J WT (stock# 000664) control mice. The genotypes were verified using PCR on tail biopsies and mice (n = 7, 24.3±1.6 grams for KO; n = 10, 26±1.8 grams for WT) were subjected to joint loading using a protocol similar to that described by Poulet *et al*. [18]. Briefly, the right lower limb was placed with the knee flexed into a Bose LM1TestBench mechanical testing system and subjected to a dynamic compression regimen over five alternate days (Fig 1). The loading regimen consisted of 80 cycles in which the waveform for each cycle lasted 5sec (Fig 1B). The load level and duration of load applied in this regimen were shown to be sufficient to induce focal cartilage lesions that progress in their extent overtime [18], but were unlikely to induce microdamage in the underlying bone epiphysis and tibial diaphysis in healthy young animals, which is typically associated with fatigue loading [19–21]. A finite element analysis performed on the tibial epiphysis under a peak load equivalent to the one used for *in vivo* loading (see below) clearly demonstrated that microdamage due to local material failure is unlikely under our loading condition with intact meniscus (data not shown). Using a separate set of animals, a single-element strain gauge (EA-06-015DJ-120; Measurements Group, Inc., Raleigh, NC) was fixed on the relatively flat anterior-medial surface (30%-50% distal to the tibial proximal end) of tibiae from both null and WT mice after sacrifice. The intact tibia was axially compressed with a gradually increasing load (0.9N/s from 0.2N to 9.2N); the resulting voltage change was measured in real-time using Bose’s data acquisition circuits. The voltage-to-strain calibration was done with aluminum cantilever beams using beam theory to derive strains [22]. The strain gauging analysis revealed no significant difference between the rigidity of the null versus WT tibiae (269.3+34.8 vs. 265.4+42.1με/N, p>0.05). Therefore, peak load of 8.5N was chosen because limbs of both genotypes could withstand comparable strain (2260–2290με) without activation of a differential woven bone response.

### Histological analysis of the knee joints

Mice were euthanized three weeks after the final loading bout. Joints were fixed in buffered-zinc formalin (Anatech Ltd., Battle Creek, MI) for 24hrs, then decalcified using formic acid-containing EDTA (Decal Chemical Corporation, Tallman, NY) for seven days, under shaking. Six micron-thick coronal knee paraffin sections were stained with Safranin-O and Fast-Green and scores were obtained from 12–15 sections/knee. The scoring was done by two independent blinded scorers in the four knee compartments using a modified semi-quantitative scoring system previously described [23, 24]. Briefly, 0 represents a normal cartilage surface, 0.5 for the loss of Safranin O with no fibrillations, 1 for the presence of minute fibrillations, 2 for fibrillations across the superficial lamina, 3 for fibrillations extending to less 20% of the entire cartilage, 4 for fibrillations extending between 20–80% of cartilage thickness. Box and whiskers graph was plotted with the average of the scores obtained from each of the four compartments across the entire knee joint.

### *In vitro* osteoblast and chondrocyte studies

Primary chondrocytes were obtained from 3–5 day-old WT mouse sterna using a protocol adapted from Dr. Lyons’ lab (UCLA, CA) and passages 1–3 were cultured in a chondrogenic media composed of DMEM supplemented with 10% (v/v) FBS and 1% (v/v) penicillin/streptomycin, 50μg/ml ascorbic acid and 10mM β-glycerophosphate. To maintain their chondrocytic phenotype, cells were cultured either as micromasses (for mRNA extraction), pellets (for immunohistochemistry), or monolayer (for alizarin red staining). MC3T3-E1, osteoblast-like cells were grown in α-MEM media supplemented with FBS and penicillin/streptomycin. Cells were subjected to FSS for 2hrs in all the experiments using a rocker platform operated at a speed of 120 RPM. The characteristic shear force experienced by cells in the center of a T-25 flask containing 5ml of media was estimated to be ~3.5 dynes/cm^2^ using Zhou *et al*. mathematical equation [25]. This system was chosen because it can achieve temporary oscillations in a sterile environment while avoiding dilution of soluble factors released in CM. In addition, it provided a system amenable for calculating the actual shear forces applied to the surface of cells (a feature that could not be achieved when cells were subjected to a circular and swaying flow motion). Our group’s recent work indicated that the peak fluid shear in bone under physiological loading conditions can be as high as 20–50 dynes/cm^2^ [26]. One limitation of the *in vitro* system used in this study is that the fluid shear stress experienced by cells using the shaker set up may be less than what cells experience *in vivo*. We predict, however, that the increase in local cell-substrate contacts *in vivo* relative to the open *in vitro system* local results in comparable strains at the cell membrane surface in these two systems.

Selective T-VSCC inhibition was achieved by pre-treating the cells with a selective T-VSCC antagonist, the NNC blocker (NNC 55–0396, Sigma-Aldrich, St Louis MO) [17]. Primary WT chondrocytes and MC3T3-E1 osteoblasts were independently grown and subjected to one of the following four conditions: 1) FSS untreated (FSS UT), 2) FSS pre-treated with NNC (FSS NNC), 3) static untreated (static UT), and static pre-treated with NNC (static NNC). Optimal time points for measuring rapid and secondary cellular responses to mechanical stress were predetermined based on our experience and the use of various experimental designs including immunocytochemical (F-actin fiber stress formation), Q-PCR and ELISA approaches as well as data from the literature [7, 11, 27]. Consequently, collection of cell extracts was performed after 2hrs of FSS followed by either a 2hr- or 20hr-rest period for assessment of early and late shear response genes, respectively. For indirect co-culture, each chondrocyte micromass was treated with CM of MC3T3-E1 subjected to one of the four conditions described above and collected after a 2hr-rest period. Treatment of micromasses tested the effect of osteoblast-derived factors released in response to mechanical stress *via* exocytosis and was performed for seven days to induce chondrocytic changes at both transcriptional and translational levels [7].

### Quantitative RT-PCR

RNA was extracted from either MC3T3-E1 monolayers or primary chondrocytes cultured as micromasses using the RNAeasy kit (Qiagen, Valencia, CA) and used as a template for cDNA synthesis (BioRad, Hercules, CA) following DNAse treatment. PCR reactions were run using the ABI 7300 PCR system (Life Technologies, Grand Island, NY) and fold changes were calculated after normalizing the data with the GAPDH housekeeping gene as described [23].

### Immunohistochemistry

Type X collagen immunodetection was performed on 12μm-thick cryosections obtained from chondrocyte pellets treated with the MC3T3-E1 CM for one week. Cryosections were subjected to a one-hour antigen exposure step via a 0.2% (w/v) Type IV-S hyaluronidase (Sigma-Aldrich, St. Louis, MO) prior to indirect immunofluorescent labeling with an anti-collagen X antibody generously provided by Dr. Horton (Shriners, Portland, OR) [23].

### Western blotting

Total cell lysates were collected 2hrs after FSS using RIPA buffer including a protease inhibitor cocktail mix (Thermo Scientific, Rockford, IL). Protein extracts were diluted 1:1 with Laemmli sample buffer, denatured at 95°C, and run into a 4–12% Bis-Tris NuPage Polyacrylamide gel at 160V for 1hr using a 1×MES SDS Running Buffer (Invitrogen, Carlsbad, CA). Proteins were then transferred to a nitrocellulose membrane using wet transfer at 40V for 4hrs and the blot was blocked overnight at 4°C in 5% (w/v) milk in TBS-Tween 20 and the blot was incubated with primary antibody against COX2 (Santa Cruz, Dallas, TX) for 2hrs. A secondary donkey horseradish peroxidase anti-goat IgG antibody was then used (Abcam, Cambridge, MA) to react with the COX2 antibody and detection was performed using a SuperSignal West Dura Chemiluminescent Substrate kit (Pierce, Thermo Scientific). The experiment was repeated three times and ImageJ software (NIH, Bethesda, MD) was used to perform densitometry.

### Statistical analysis

The histological scores were analyzed using a non-parametric *Kruskall-Wallis* test and *Bonferroni* correction was performed for multiple comparisons as described [28]. Gene expression fold changes and Western blot densitometric values were analyzed using a *Student* t-test and *p* values below 0.05 were considered significant. All *in vitro* experiments were performed using cell lines issued from different batches or different animals and the data are presented as the mean of at least six individual values obtained from experiments run at different times.

## Results

### Ca_v_3.2 T-VSCC KO mice display reduced cartilage damage relative to WT controls following repetitive knee loading

**Fig 2:**
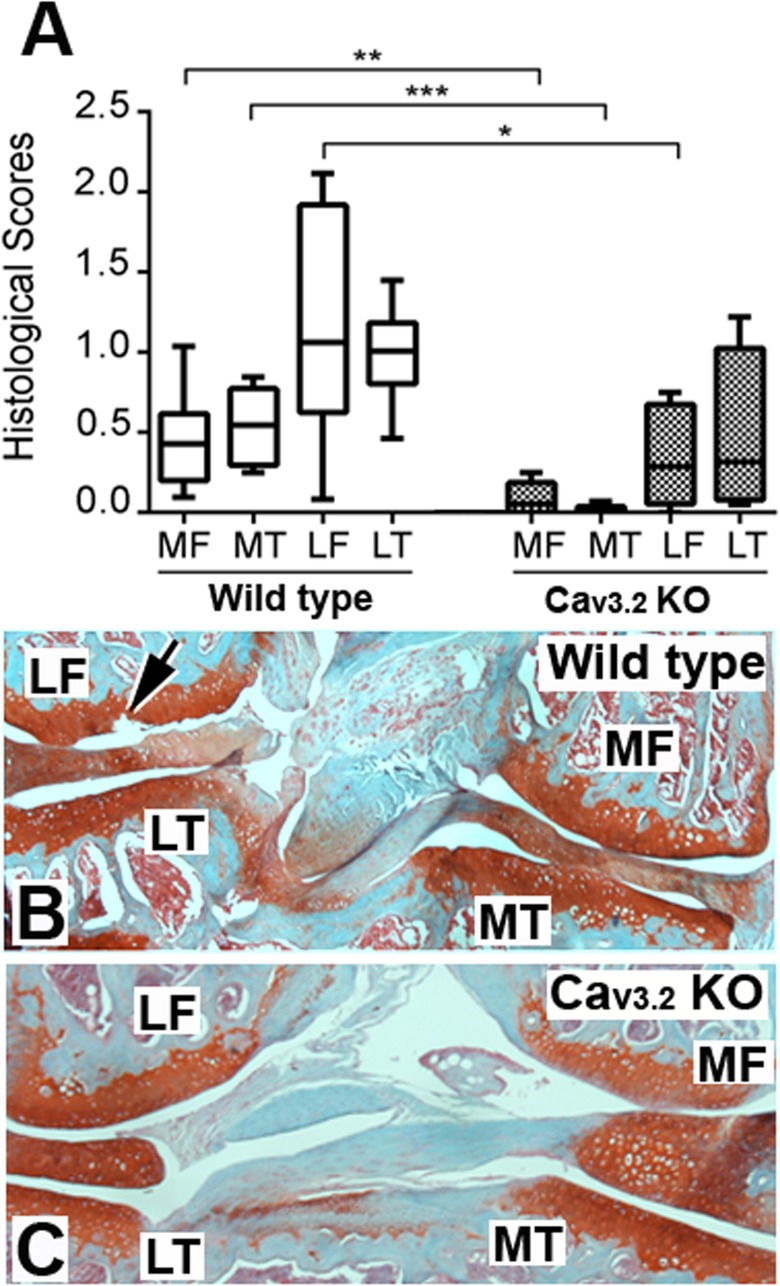
Decrease of OA damage in T-VSCC KO vs. WT controls following *in vivo* knee loading. A: boxes and whiskers graph showing the median (central line), 25–75% (box) and the entire range (whiskers) of average histological OA scores obtained three weeks after final loading in the four compartments (MT-Medial Tibia, MF- Medial Femur, LT-Lateral Tibia, LF- Lateral Femur) of wild type control and T-VSCC KO mouse knees. B and C are coronal knee sections stained with Safranin O and Fast green from either a WT or a T-VSCC KO loaded knee, respectively. Arrow in B indicates a focal lesion caused by loading in the lateral knee compartment of a WT mouse knee. * p = 0.012, ** p = 0.027 and *** p = 0.001; n = 10 for wild type and n = 7 for T-VSCC KO mice. An average of 15 slides representative of the entire knee were blinded and scored by two independent observers using scoring system modified from Glasson *et al* [24].

To determine whether a link exists between mechanical stimulation through T-VSCC activation and OA, we subjected age-matched Ca_v_3.2 T-VSCC KO and WT mice to repetitive knee loading for five alternate days. Histological analysis of sections of loaded knees harvested three weeks after *in vivo* loading showed that KO knees had significantly lower mean OA scores than the WT control knees (Fig 2; p = 0.001). Furthermore, the comparison of OA scores within individual knee compartments (Fig 2A) showed that the loaded KO mouse sections had significantly lower OA scores in the medial femur (p = 0.012), medial tibia (p = 0.001), and lateral femur (p = 0.027) than in the corresponding compartments of loaded WT control knees. The maximum OA damage was observed in the lateral femur of WT mice (see Fig 2A and arrow in Fig 2B). Such focal damages were primarily found in the posterior and lateral positions of WT knee especially in the lateral femur compartment where contact loading force experienced by the articular cartilage surface is maximal.

### T-VSCC is required for the anabolic response of osteoblasts to FSS

**Fig 3:**
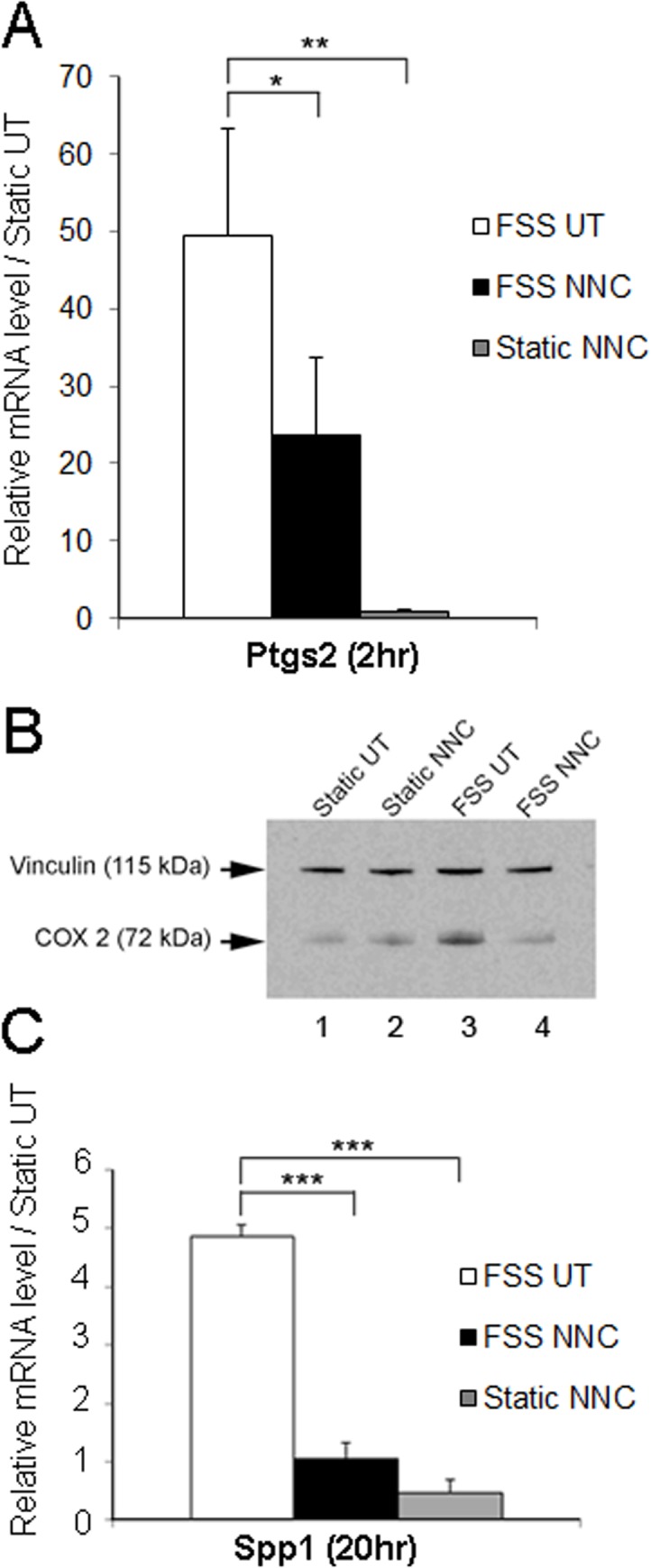
The fluid shear stress (FSS)-induced expression of the early shear response marker cyclooxygenase 2 and the late shear response shear response gene osteopontin are inhibited in MC3T3-E1 cells with the addition of T-VSCC-specific inhibitor, NNC55-0396 (NNC). **A**: quantitative PCR analysis shows that the marked increase in Cox2 (Ptgs2) mRNAs observed 2hrs following FSS relative to the static untreated (Static UT) control condition is significantly inhibited in the presence of NNC. The conditions are FSS untreated (FSS UT), FSS treated with NNC (FSS NNC), static treated with NNC (static NNC). Error bars represent standard error of mean of biological duplicates and * indicates p = 0.026 between FSS UT and FSS NNC, ** p = 0.006 between FSS UT and static NNC. **B**: western blot analysis performed under the same conditions described in A indicates that the FSS-induced increase of COX2 protein is decreased to control levels in the presence of NNC. Vinculin was used as a loading control. **C**: Quantitative PCR analysis shows that the increase in osteopontin (Spp1) mRNAs observed 20hrs following FSS relative to the static untreated (Static UT) control condition is significantly inhibited in the presence of NNC. The conditions are FSS untreated (FSS UT), FSS treated with NNC 55–0396 (FSS NNC), static treated with NNC (static NNC). Error bars represent standard error of mean of biological duplicates and *** p<0.0001 between FSS UT and FSS NNC or FSS UT and static NNC.

To examine changes in shear-response genes indicative of an early and late anabolic response in bone, MC3T3-E1 osteoblasts were exposed to FSS for 2hrs and RNA was extracted 2hrs following mechanical stimulation (Fig 3A). A nearly 50-fold increase was found for Ptgs2 (Cox2) mRNA levels in cells subjected to FSS (FSS UT) relative to static control cells (static UT) within 2hrs following FSS (Fig 3A). This increase in Ptgs2 transcripts was accompanied by a marked increase in COX2 protein levels (Fig 3B, lane 3). In addition, this early response was followed by an approximate 5-fold increase in osteopontin (Spp1) mRNA levels in FSS vs. static control cells 20hrs after FSS stimulation (Fig 3C). Ptgs2 and Ssp1 mRNA level increases were significantly reduced when MC3T3-E1 cells were subjected to FSS in the presence of NNC (FSS NNC). In contrast, cells maintained under static conditions and subjected to NNC treatment (Static NNC) showed no significant changes in Ptgs2 or Spp1 levels when compared with static control cells. Western blot analysis comparing COX2 protein levels among these four experimental conditions in osteoblasts showed that the COX2 protein increase in response to FSS is also attenuated to control levels by the T-VSCC blocker (Fig 3B, lane 4).

Primary osteoblasts isolated from WT mice exhibited a much lower response after 2hrs of FSS treatment relative to MC3T3-E1 cell lines, with only a 2-fold increase in Ptgs2 mRNA levels vs. static conditions (S1 Fig). Interestingly, the inhibition of T-VSCC function in primary KO osteoblasts produced changes similar to that observed in MC3T3-E1 cells treated with the T-VSCC inhibitor and Ptgs2 mRNAs were decreased in FSS KO osteoblasts vs. FSS WT osteoblasts (S1 Fig). Nonetheless, all further studies were conducted using the MC3T3-E1 cell line in order to produce a maximal response and avoid limitations related to suboptimal concentrations of factors released by osteoblasts.

### T-VSCC inhibition reverses the chondrocyte hypertrophy induced by the CM from FSS osteoblasts

**Fig 4:**
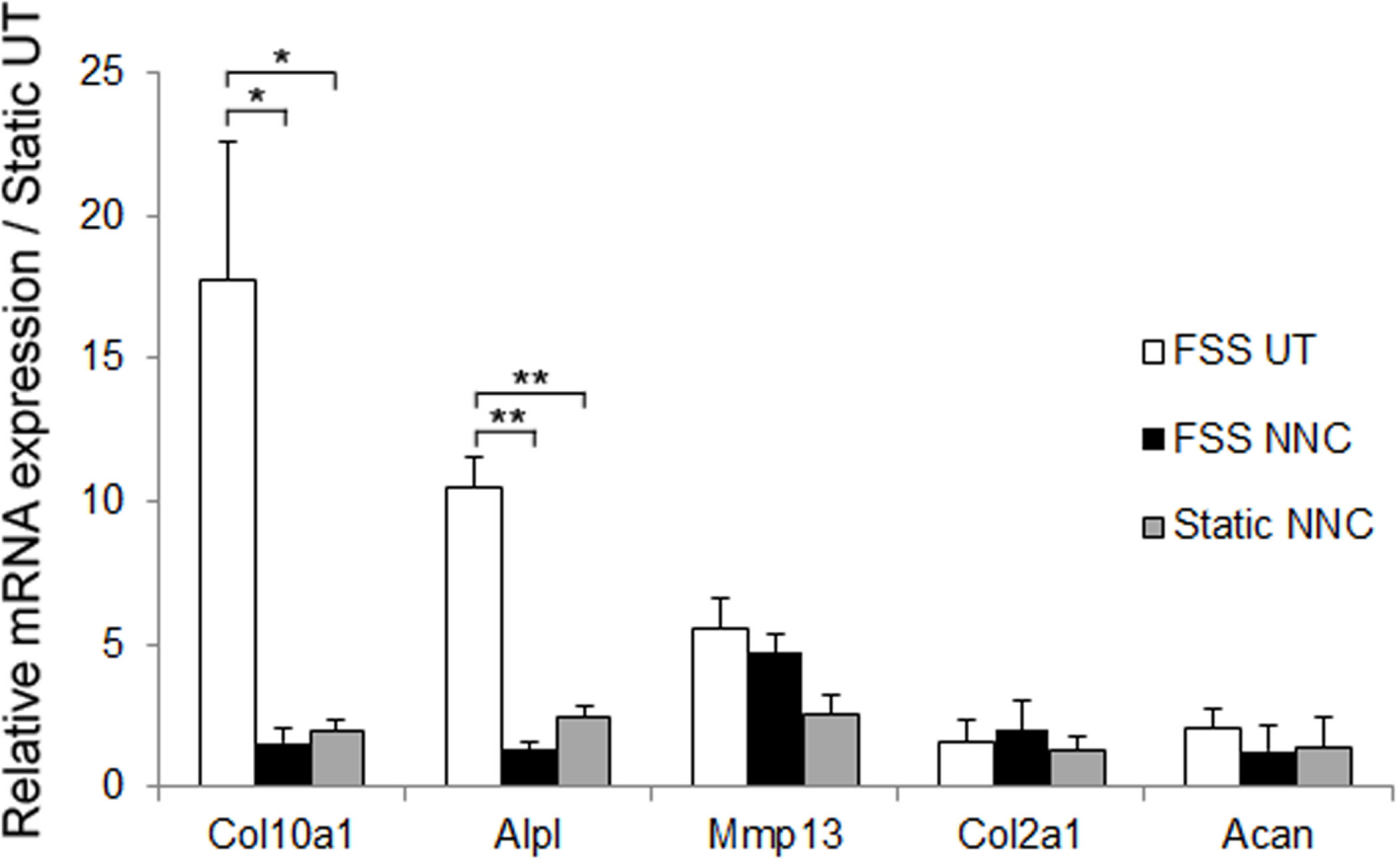
The early OA-like phenotype of chondrocytes is attenuated when treated with conditioned media derived from osteoblast sheared in the presence of the T-VSCC inhibitor, NNC55-0396 (NNC). A: quantitative PCR showing the relative fold changes in mRNA levels of collagen X (Col10a1), alkaline phosphatase (Alpl), Matrix Metalloproteinase 13 (Mmp13), aggrecan (Acan), and collagen II (Col2a1) in primary mouse chondrocytes following seven days of treatment with CM collected from MC3T3-E1 cells subjected to 2hrs of FSS alone (FSS UT), 2hrs of FSS with NNC (FSS NNC) or static with NNC (Static NNC) when compared with untreated control chondrocytes maintained under static conditions (Static UT). Error bars represent standard error of mean of biological duplicates and * p = 0.04, ** p = 0.01.

To determine whether mechanically-challenged osteoblasts can trigger an OA-like phenotype in chondrocytes using our *in vitro* system, we obtained osteoblast-derived CM from osteoblasts cultured under either static or FSS conditions and compared the expression profiles of primary mouse chondrocyte micromasses treated with such osteoblast-derived CMs (FSS UT vs. Static UT, Fig 4). In addition, the contribution of T-VSCC function was examined by adding NNC to the medium during FSS and testing the effect of the resulting CM (FSS NNC) on chondrocytes. The CM from sheared osteoblasts (FFS UT) induced up-regulation of collagen X (Col10a1), alkaline phosphatase (Alpl), and Mmp13 mRNAs in chondrocytes relative to CM obtained from static control cells (Fig 4). In contrast, chondrocytes cultured with CM obtained from both sheared (FSS NNC) or non-sheared (Static NNC) osteoblasts cultured in the presence of NNC displayed a significant reduction in the expression of both *Col10a1* and *Alpl* genes relative to the FSS UT condition. Although there were no significant differences in the levels of aggrecan and collagen II across the various conditions, we found a decreasing trend in the expression level of Mmp13 mRNA in chondrocytes treated with CM from FSS NNC and static NNC *vs*. chondrocytes treated with CM from FSS UT (Fig 4).

**Fig 5:**
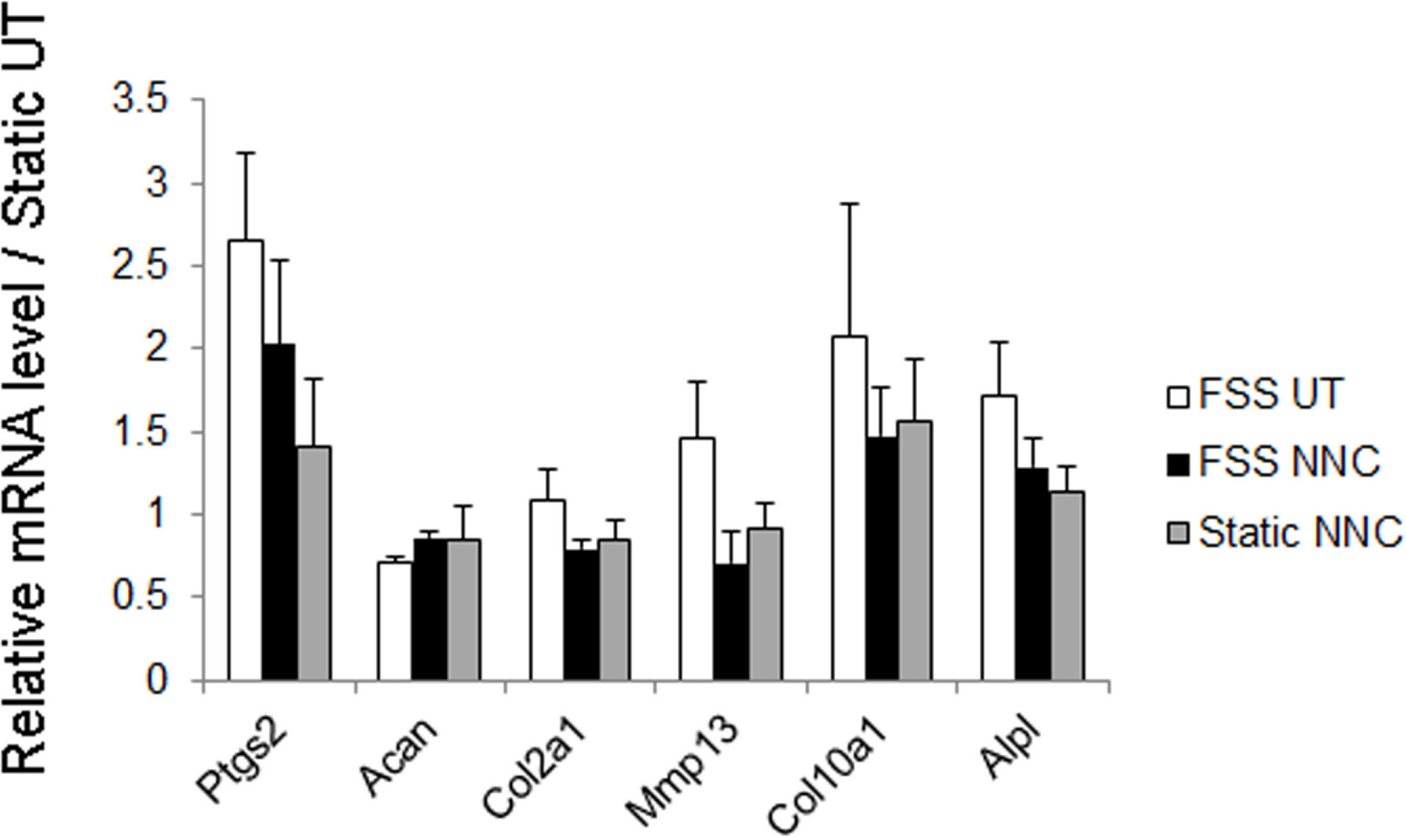
FSS triggers a response in chondrocytes that is reversed in the presence of NNC55-0396 (NNC) without altering the expression of transcripts encoding for markers of cartilage ECM. Real time PCR analysis showing the relative fold changes in the mRNA levels of cyclooxygenase 2 (ptgs2), collagen X (Col10a1), matrix metalloproteinase 13 (Mmp13), aggrecan (Acan), collagen II (Col2a1), and alkaline phosphatase (Alpl) in primary mouse chondrocytes grown in monolayer and collected 20hrs after FSS alone (FSS UT), FSS with NNC (FSS NNC) or maintained under static conditions with NNC (Static NNC) compared with the untreated static control condition (Static UT). The error bars represent standard error of mean of biological duplicates.

The direct effect of FSS and/or T-VSCC inhibitor on primary mouse chondrocytes was tested using the same conditions described above for osteoblasts (Fig 5). FSS increased the relative levels of Ptgs2 transcripts in chondrocytes when compared to static untreated (Static UT) chondrocytes, albeit to a lower extent than what was observed in MC3T3-E1 cells. Addition of NNC to the medium during FSS decreased *Ptgs2* gene activation to near basal levels (Static UT). Interestingly, the expression of the *Mmp13* gene, a marker of cartilage matrix degradation, showed a slight elevation following shear stress, but also returned to basal levels in the presence of the T-VSCC inhibitor. In contrast, no clear direct effect was observed on either *Col10a1* or *Alpl* gene expression upon NNC administration (FSS NNC) relative to FSS alone (FSS UT) or static controls. Importantly, no detrimental effect on cartilage matrix marker gene expression such as collagen II (*Col2a1*) and aggrecan (*Acan*) was observed when chondrocytes were cultured in the presence of NNC (Static NNC or FSS NNC). In fact, a small but significant (p = 0.022) increase in Acan mRNA was found in FSS NNC *vs*. FSS UT (Fig 5).

**Fig 6:**
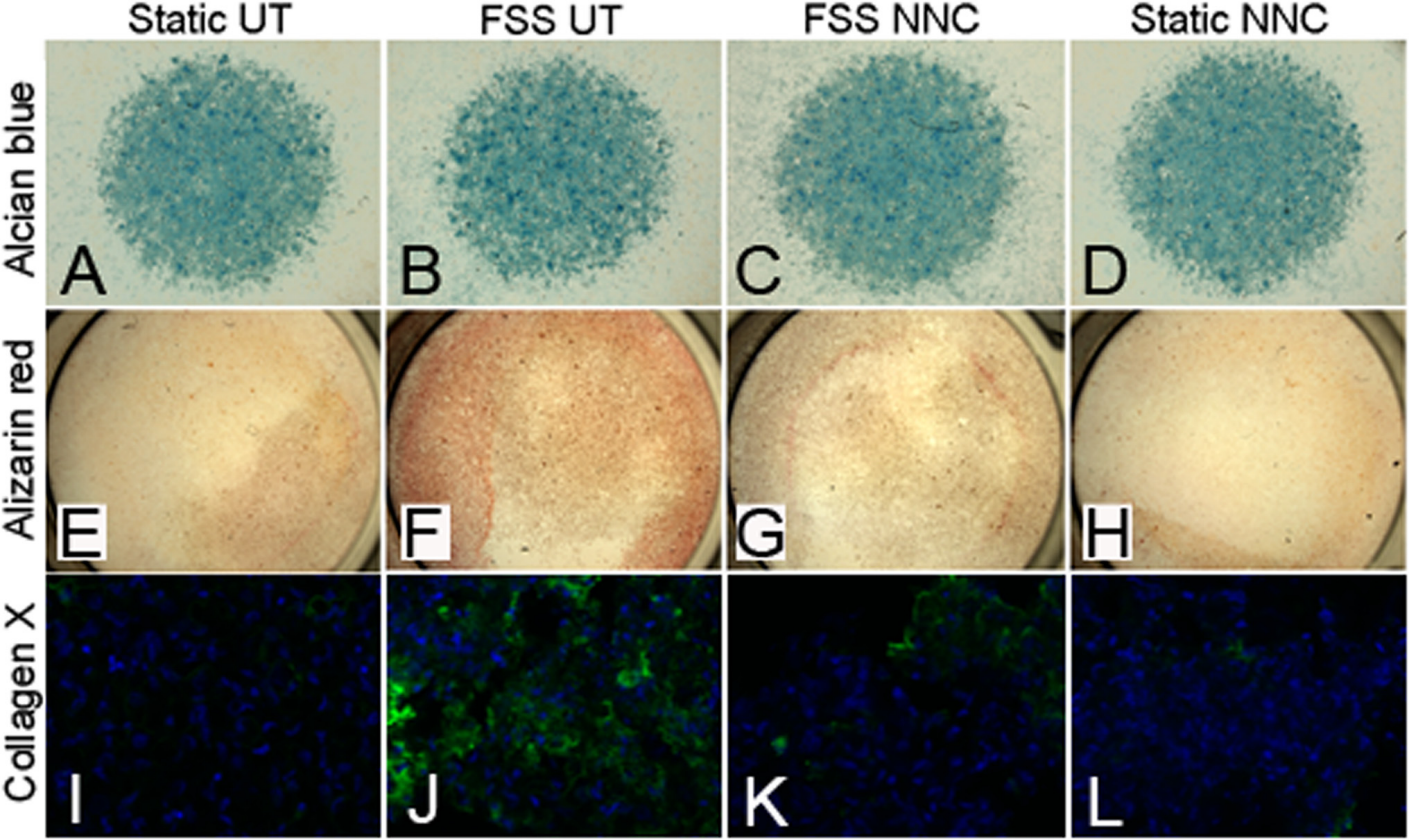
The early OA differentiation phenotype observed in primary chondrocytes treated with CM from FSS-stimulated osteoblasts is prevented in the presence of the T-VSCC inhibitor, NNC55-0396 (NNC). Primary mouse chondrocytes grown in micromasses or as monolayers and stained with Alcian blue (A-D), Alizarin red (E-H) or collagen X antibody (I-L), respectively. Staining was performed following seven days of treatment with CM collected from MC3T3-E1 cells subjected to FSS alone (FSS UT), FSS with NNC (FSS NNC), static with NNC (static NNC) or control media obtained from MC3T3-E1 cells maintained under static conditions (Static UT). Whereas no significant difference is observed among the conditions with Alcian blue, the Alizarin red and collagen X stainings (green-collagen X, blue- DRAQ5 nuclear stain) showed an obvious increase in calcium deposits (F) and collagen X-specific signal (J) among chondrocytes treated with CM from FSS UT and compared with chondrocytes treated with CM from FSS NNC and both static conditions (Static UT and Static NNC).

To ascertain whether the changes in mRNA transcript levels reflect a phenotypic change in chondrocytes, we performed 1) alcian blue staining for sulfated glycosaminoglycans, 2) alizarin red staining for assessment of the presence of calcium deposits associated with a switch to hypertrophy/ectopic calcification, and 3) collagen X immunohistochemical staining for a direct measure of hypertrophy. Whereas alcian blue staining showed no noticeable difference among the four conditions tested (Fig 6A–6D), alizarin red and collagen X stainings depicted increased staining in chondrocytes treated with CM from sheared osteoblast (Fig 6F and 6J) compared to those treated with CM obtained from osteoblasts sheared in the presence of NNC (Fig 6G–6H and 6K–6L) and the static control condition (Fig 6E and 6I).

## Discussion

It is well accepted that abnormal metabolic activity associated with elevated bone mineral density and increased subchondral bone sclerosis play a key role as predictors of OA [29, 30]. Here, we propose that reduced mechanosensitivity *via* inhibition of the T-VSCC is beneficial in preventing early stage OA and used an *in vivo* gene-targeting strategy and an *in vitro* inhibitor approach to test this idea. Recent work showed that Ca_v_3.2 T-VSCC KO mice exhibit reduced bone formation and bone remodeling properties following disuse-induced bone loss, suggestive of a major role for the Ca_v_3.2 T-VSCC in bone mechanotransduction [15]. This phenotype indicates that the Ca_v_3.2 T-VSCC null mouse model is ideal to study the *in vivo* effect of reduced mechanoresponsiveness on OA development. Although, the absence of functional Ca_v_3.2 T-VSCC in these mice did not alter the overall integrity of the cartilage, Ca_v_3.2 KO mice exhibited significantly less focal cartilage damage following repetitive knee loading compared to age-matched WT control mice. Since increased bone mineral density and bone turnover are strongly associated with OA, the observed results suggest that the reduced bone remodeling properties in the Ca_v_3.2 KO mice might be beneficial in preventing OA pathogenesis [1, 2, 31].

Several ion channels have been associated with the mechanical response of osteoblasts *in vitro* and bone formation in *vivo*, including the Ca_v_1.2 L-type VSCC[32, 33], mechanosensitive cation-selective channel (MSCC) [32, 34] and the transient receptor potential vanilloid 4 channel (TRPV4) [35]. One that is of particular interest is the TRPV4 channel which has been found in both cartilage [36] and bone [37]. This channel is known to be important in normal cartilage development [36, 38] and chondrocyte’s response to mechanical stimulation [39]. Recent study performed in mice deficient for TRPV4 showed progressive cartilage degeneration in aging Trpv4^-/-^ mice relative to WT controls, indicative of its chondroprotective role [40]. Interestingly, development of spontaneous OA in Trpv4^-/-^ mice occurs in association with increased subchondral bone thickness and mineralization of the meniscal tissues. This is consistent with our findings seen in the Ca_v_3.2 KO mice, where decreased subchondral bone remodeling is associated with chondroprotection.

To delineate the role of the T-VSCC in osteoblast mechano-transduction of downstream signaling events, MC3T3-E1 osteoblastic cells were subjected to FSS with or without an inhibitor that prevents calcium currents by interfering with the gating of the T-VSCC pore-forming subunit. The observed reduction in the expression of stress-response genes such as COX2 and osteopontin following the inhibition of T-VSCC indicates that T-VSCC activity is essential for the anabolic bone response to load and is in agreement with decreased calcium signaling and the reduced bone formation rate observed in Ca_v_3.2 KO mice [15]. Previous studies demonstrate a positive relationship between subchondral bone volume and OA and indicate that bone adaptation to altered mechanical loading is associated with the release of bone-derived anabolic markers and cytokines [6, 41–43]. We next sought to determine whether factors released by mechanically stimulated osteoblasts can alter the chondrocyte phenotype *in vitro* and if T-VSCC inhibition in osteoblasts can attenuate the resulting pro-catabolic response on chondrocytes. The chondrocyte-osteoblast co-culture model was chosen to expand on our *in vivo* study mainly because bone and cartilage are the primary joint tissues exposed to external compressive loading *via* direct contact between femoral condyles and the tibial plateau and osteoblasts are the primary cells responsible for subchondral bone deposition. In addition, osteoblasts and osteocytes belong to the same cell lineage and are both sensitive to mechanical loading [44, 45]. More importantly, this work supports a direct role for T-VSCC and its associated calcium response to shear stress in osteoblasts and is consistent with previous T-VSCC blocker studies performed using primary bone cell cultures and an osteocytic cell line [15, 46].

As expected, the CM obtained from shear-stressed osteoblasts induced a hypertrophic phenotype in primary mouse chondrocytes. Up-regulation of collagen X and alkaline phosphatase expression by chondrocytes is indicative of hypertrophy and premature calcification, respectively; and is associated with the initial steps of OA that are generally followed by activation of a large panel of cartilage matrix degrading enzymes including MMPs [7, 47, 48]. Thus, the current data suggest that biochemical factors released from osteoblasts in response to mechanical load induce an early OA phenotype in chondrocytes. Prasadam, et al. showed, using the same co-culture system, that media derived from osteoblasts obtained from OA patients induced a similar hypertrophic response in normal articular chondrocytes and significantly decreased the expression of cartilage-specific genes [7]. We were not able to observe down-regulation of cartilage-specific genes using CM derived from sheared healthy osteoblasts, which may point to signaling differences between young healthy and aged, diseased cells. Regardless, expression of both hypertrophic markers was significantly attenuated in chondrocytes treated with CM derived from sheared osteoblasts pre-treated with the T-VSCC-specific inhibitor, supporting the involvement of T-VSCC in the hypertrophic response of chondrocytes from bone-derived factors.

The treatment of FSS osteoblasts with the T-VSCC inhibitor did not return the expression of COX2 to static control levels. Nonetheless, subsequent treatment of chondrocytes with CM obtained from FSS NNC-treated osteoblasts reduced the levels of hypertrophic genes to baseline. This is likely because other factors in addition to prostaglandins are secreted from sheared osteoblasts as a result of load-induced T-VSCC activation. Although identifying specific bone-derived factors released upon loading of subchondral bone is beyond the scope of this paper, the results presented in this study clearly show that the co-culture FSS system is an attractive experimental system for the discovery of novel metabolites with catabolic activity on chondrocytes. As seen in this study, COX2 followed by osteopontin were also increased in human OA joints along with alkaline phosphatase activity [49, 50]. Even though this raises an obvious strategy for targeting a factor such as COX2 or a specific cytokine in OA patients, the ineffectiveness of such single factor strategies is evident from the failure of the COX inhibitors in reversing the natural history of the disease. Thus, it will be more effective to block an upstream target like T-VSCC rather than the individual downstream players, as this approach would result in impaired calcium signaling and diminished secretion of multiple bone-derived factors with pro-catabolic activity on cartilage.

To determine if chondroprotection in KO mice is due to the lack of T-VSCC in bone cells or chondrocytes, we also subjected the murine primary chondrocytes to FSS with or without a T-VSCC specific inhibitor. Direct mechanical stimulation of chondrocytes produced a minimal response when subjected to the same FSS conditions as bone cells. Even though the observed effect occurred to a much lower extent in chondrocytes than in osteoblasts, it is noteworthy to mention that the treatment with T-VSCC inhibitor had no negative side effect on cartilage matrix markers and even resulted in a slight increase in aggrecan gene expression. This suggests that the blocking of T-VSCC may in fact have some direct favorable effect on cartilage in addition to inhibition of osteoblast-induced hypertrophic differentiation. Based on the differential response of osteoblasts and chondrocytes to FSS with T-VSCC inhibition *in vitro*, we conclude that the reduced cartilage damage in T-VSCC null mice results predominantly from the lack of T-VSCC function in osteoblasts rather than in chondrocytes.

Although, T-VSCC is not the only pathway for initial calcium influx in mechanically-loaded joint tissues [46], the current work clearly reveals that T-VSCC plays a direct role in cellular events associated with mechanical regulation of bone tissue and is subsequently responsible for inducing an early OA phenotype, which may eventually lead to cartilage thinning and loss. We believe that blocking of T-VSCC will reduce the bone changes and pro-inflammatory cytokine production which, in turn, is important to maintain cartilage integrity. Previous tracer studies using small molecular weight fluorescent markers of similar size as the T-VSCC inhibitor (NNC 53–0396, 565Da) indicate that direct communication exists between articular chondrocytes and subchondral osteoblasts, particularly in osteoarthritic joints [42]. Therefore, the direct continuation of this work will be to test the chondroprotective effect of T-VSCC inhibitor using local intra-articular injections in preclinical models of OA. As with any intra-articular injections and drug delivery systems, rapid clearance and degradation of the injected T-VSCC inhibitor are potential problems [23, 51]. This issue can be counteracted by conjugating T-VSCC inhibitor with hyaluronan-based microgels for time-dependent controlled delivery [23]. Although disruption of T-VSCC function was found to interfere with the normal relaxation of coronary arteries [12], local administration of the T-VSCC inhibitor using a slow release delivery system will prevent systemic circulation of the drug and limit risk of side effects. Hyaluronan (HA)-based macromolecules are commonly used in the clinic as viscosupplements to enhance joint mobility and provide temporary relief of knee pain by increasing the viscosity of synovial fluid but do not block disease progression. Thus, the combined injection of HA along with an agent such as the T-VSCC inhibitor that slows the disease progression would certainly provide a superior therapeutic option to patient suffering from OA. One additional advantage to using a T-VSCC inhibitor for OA emerges from its role in nociception. In particular, Ca_v_3.2 subunit has been implicated in modulating chronic peripheral and central pain. Indeed, the target of several novel analgesic drugs is Ca_v_3.2 T-VSCC and local injection of a T-VSCC inhibitor may therefore be an effective treatment for OA and an alternative for systemic NSAIDS [52–54].

## Conclusions

In summary, this study indicates that T-VSCC is a novel mediator of OA progression by increasing load-induced signaling between subchondral osteoblasts and articular chondrocytes. These data further suggest that the T-VSCC may be a potential target for prevention of OA progression and selective T-VSCC blockers such as NNC could be used in the treatment of load-induced OA. Future translational investigations will focus on establishing T-VSCC inhibitors as locally administered DMOAD for lessening cartilage and subchondral bone OA changes in both post-traumatic and age-related OA.

## Supporting Information

S1 Fig The FSS-induced expression Cox2 is inhibited in T-VSCC KO osteoblasts *vs*. WT osteoblasts. Quantitative PCR analysis using primary osteoblasts shows that the increase in Cox2 (Ptgs2) mRNAs observed 2hrs following fluid shear stress in WT osteoblasts (WT FSS) relative to the control condition is inhibited in T-VSCC KO osteoblasts. The conditions are as follows: WT osteoblasts static (WT Static), WT osteoblasts FSS-stressed (WT FSS), T-VSCC KO osteoblasts static (KO Static), and T-VSCC KO osteoblasts FSS-stressed (KO FSS). Error bars represent standard error of mean of biological duplicates.
(TIF)

S1 Method Primary osteoblast isolation. (DOCX)
