# Supplementary figures and images for: Inhibition of T-Type Voltage Sensitive Calcium Channel Reduces Load-Induced OA in Mice and Suppresses the Catabolic Effect of Bone Mechanical Stress on Chondrocytes
Source: PLoS One. 2015 May 26;10(5):e0127290. doi: 10.1371/journal.pone.0127290 (PMC4444170; doi:10.1371/journal.pone.0127290)

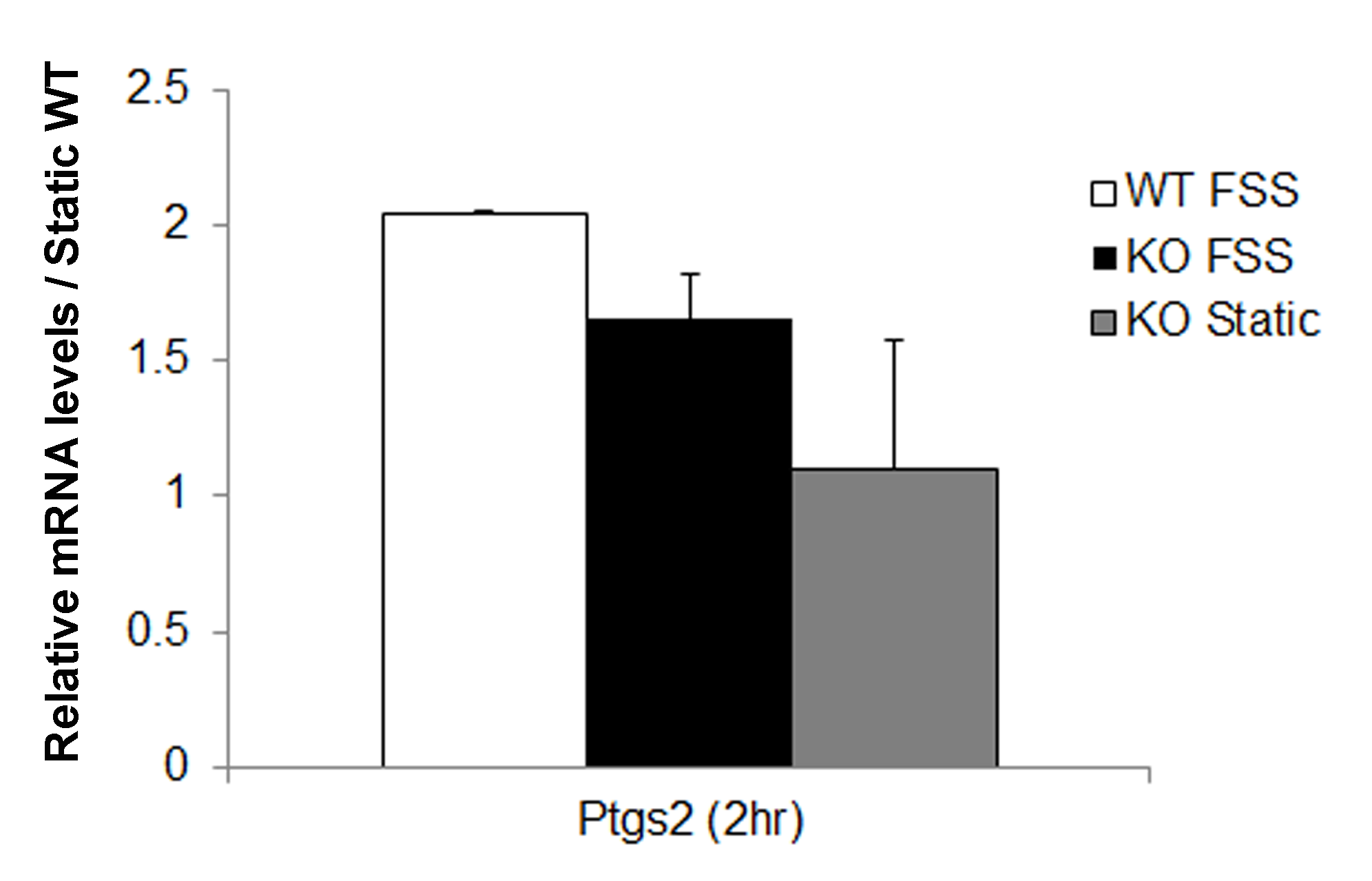

Supplement: S1 Fig — Quantitative PCR analysis using primary osteoblasts shows that the increase in Cox2 (Ptgs2) mRNAs observed 2hrs following fluid shear stress in WT osteoblasts (WT FSS) relative to the control condition is inhibited in T-VSCC KO osteoblasts. The conditions are as follows: WT osteoblasts static (WT Static), WT osteoblasts FSS-stressed (WT FSS), T-VSCC KO osteoblasts static (KO Static), and T-VSCC KO osteoblasts FSS-stressed (KO FSS). Error bars represent standard error of mean of biological duplicates. (TIF) [file pone.0127290.s001.tif]
