# Supplementary material for: Inhibition of T-Type Voltage Sensitive Calcium Channel Reduces Load-Induced OA in Mice and Suppresses the Catabolic Effect of Bone Mechanical Stress on Chondrocytes
Source: PLoS One. 2015 May 26;10(5):e0127290. doi: 10.1371/journal.pone.0127290 (PMC4444170; doi:10.1371/journal.pone.0127290)
Supplement: S1 Method — (DOCX) [file pone.0127290.s002.docx]

**S1 Method. Primary osteoblast isolation.** Primary mouse osteoblasts were extracted from the cranium of 3-5 day-old C57BL/6J wild type and T-VSCC KO pups, cleaned under microscope in 1× PBS solution and were placed in the medium containing DMEM (Invitrogen), 10% (v/v) FBS (Hyclone) and 1% (v/v) Penn/Strep overnight. On the following day, the calvarial halves were washed in 2ml of collagenase solution (containing 5.6mM glucose, Mg^2+/^Ca^2+^-free PBS and type 1 collagenase [Worthington Biochemical Corporation, Lakewood, NJ] at 230U/ml) prepared in 1×PBS and placed on a shaker at 37°C for 15min to remove residual tissues. Calvarial halves were washed again in 1×PBS and shaken for an additional 1hr in the collagenase solution. The resulting suspension containing the osteoblasts was filtered using a cell strainer and centrifuged at 1000rpm for 10min to obtain a cell pellet. The pellet was then suspended in DMEM complete media and plated into a 6-well plate. Cells were split upon reaching 80-90% confluence and passages below three were used for experiments. WT and KO osteoblasts were grown in α−MEM media supplemented with FBS and penicillin/streptomycin and subjected to fluid shear stress (FSS) for 2hrs using a rocker platform. RNA extraction was performed after 2hrs of FSS followed by a 2hr-rest period. Control WT and KO cells were grown under static conditions. Quantitative RT-PCR was performed as described for the MC3T3-E1 cell line.
